# Evaluation of novel Epstein-Barr virus-derived antigen formulations for monitoring virus-specific T cells in pediatric patients with infectious mononucleosis

**DOI:** 10.1186/s12985-024-02411-0

**Published:** 2024-06-14

**Authors:** Franziska Fischer, Johannes Mücke, Louisa Werny, Katrin Gerrer, Lorenz Mihatsch, Stefanie Zehetmaier, Isa Riedel, Jonas Geisperger, Maren Bodenhausen, Lina Schulte-Hillen, Dieter Hoffmann, Ulrike Protzer, Josef Mautner, Uta Behrends, Tanja Bauer, Nina Körber

**Affiliations:** 1 Children’s Hospital, School of Medicine, Technical University of Munich, Munich, Germany; 2 Institute of Virology, School of Medicine, Technical University of Munich and Helmholtz Munich, Schneckenburgerstr. 8, 81675 Munich, Germany; 3 Research Unit Gene Vectors, Helmholtz Munich, Munich, Germany; 4 German Centre for Infection Research (DZIF), Munich, Germany

**Keywords:** Infectious mononucleosis, Epstein-Barr virus, T-cell response, Immune monitoring, Pediatric patients

## Abstract

**Background:**

Infection with the Epstein-Barr virus (EBV) elicits a complex T-cell response against a broad range of viral proteins. Hence, identifying potential differences in the cellular immune response of patients with different EBV-associated diseases or different courses of the same disorder requires interrogation of a maximum number of EBV antigens. Here, we tested three novel EBV-derived antigen formulations for their ability to reactivate virus-specific T cells *ex vivo* in patients with EBV-associated infectious mononucleosis (IM).

**Methods:**

We comparatively analyzed EBV-specific CD4+ and CD8+ T-cell responses to three EBV-derived antigen formulations in 20 pediatric patients during the early phase of IM: T-activated EBV proteins (BZLF1, EBNA3A) and EBV-like particles (EB-VLP), both able to induce CD4+ and CD8+ T-cell responses *ex vivo,* as well as an EBV-derived peptide pool (PP) covering 94 well-characterized CD8+ T-cell epitopes. We assessed the specificity, magnitude, kinetics, and functional characteristics of EBV-specific immune responses at two sequential time points (v1 and v2) within the first six weeks after IM symptom onset (T_onset_).

**Results:**

All three tested EBV-derived antigen formulations enabled the detection of EBV-reactive T cells during the early phase of IM without prior T-cell expansion *in vitro*. EBV-reactive CD4+ and CD8+ T cells were mainly mono-functional (CD4+: mean 64.92%, range 56.15-71.71%; CD8+: mean 58.55%, range 11.79-85.22%) within the first two weeks after symptom onset (v1) with IFN-γ and TNF-secreting cells representing the majority of mono-functional EBV-reactive T cells. By contrast, PP-reactive CD8+ T cells were primarily bi-functional (>60% at v1 and v2), produced IFN-γ and TNF and had more tri-functional than mono-functional components. We observed a moderate correlation between viral load and EBNA3A, EB-VLP, and PP-reactive CD8+ T cells (*r*_*s*_ = 0.345, 0.418, and 0.356, respectively) within the first two weeks after T_onset_, but no correlation with the number of detectable EBV-reactive CD4+ T cells.

**Conclusions:**

All three EBV-derived antigen formulations represent innovative and generic recall antigens suitable for monitoring EBV-specific T-cell responses *ex vivo*. Their combined use facilitates a thorough analysis of EBV-specific T-cell immunity and allows the identification of functional T-cell signatures linked to disease development and severity.

**Supplementary Information:**

The online version contains supplementary material available at 10.1186/s12985-024-02411-0.

## Background

The Epstein-Barr virus (EBV) is a ubiquitous γ-herpesvirus that establishes lifelong persistent infection in more than 90% of the human population. EBV is usually transmitted *via* saliva and colonizes the human host by latently infecting B cells [1, 2]. While primary EBV infection during early childhood is primarily asymptomatic, delayed primary infection during adolescence or adulthood may cause infectious mononucleosis (IM), a febrile illness typically associated with pharyngitis, cervical lymphadenopathy, splenomegaly, hepatitis, and fatigue that usually resolves within one to six weeks without sequelae [3–5]. In some patients, however, acute complications can be severe or even life-threatening. These include hematological or neurological disorders in up to 50% and 5%, respectively, as well as splenic rupture or upper airway obstruction each in up to 1% of all IM patients [6]. Severe complications account for about 30 IM-associated deaths per year in the U.S. [7]. Moreover, EBV is a common trigger of hemophagocytic lymphohistiocytosis (EBV-HLH), a life-threatening manifestation of severe immune dysregulation [8, 9]. After IM, protracted symptoms such as fatigue and post-exertional malaise (PEM) can last for months or even years [5–8], and EBV is a common trigger of post-infectious myalgic encephalomyelitis/chronic fatigue syndrome (ME/CFS). Post-infectious ME/CFS is a severe and complex neurological disease known to significantly compromise daily activities, including school attendance or working ability, as well as quality of life [10, 11]. ME/CFS has been reported in 13% and 4% of adolescents 6 and 24 months after IM, respectively [7, 12]. Furthermore, a history of EBV-IM is a risk factor for the development of Hodgkin lymphoma and multiple sclerosis [13, 14], adding to the 32-fold increased risk of multiple sclerosis in any EBV-infected person [15]. Distinct primary immunodeficiencies (PID) have been identified as predisposing factors in some cases of fulminant IM [16, 17], but in most patients, the etiology of severe or protracted disease remains elusive. No causal therapy for IM exists and immunosuppression remains the mainstay of therapy for life-threatening IM [18, 19].

Immune dysregulation, including an overresponding T-cell compartment, has been invoked as responsible for the clinical symptoms. Compared to patients with IM, individuals who acquire EBV asymptomatically may show similarly high circulating viral loads and a qualitatively similar T-cell response, but the magnitude of the response is generally lower and total lymphocyte counts in peripheral blood are barely increased [5, 20, 21]. These findings imply a potential correlation of T cell-responses with IM severity and protraction. The characterization of the immune response during primary EBV infection may, therefore, identify signatures of effective antiviral immunity as well as risk-parameters for acute or late complications of IM, and possibly for distinct malignant or autoimmune diseases.

Different approaches are currently applied to assess frequency and phenotype of EBV-specific T cells *ex vivo*, foremost ELISpot and flow cytometry-based methods, including intracellular cytokine and peptide-major histocompatibilty complex (pMHC) multimer staining [22, 23]. Although considered the gold standard in monitoring EBV immunity, a major limitation of all these different approaches is the low number of antigens that can be analyzed simultaneously. Binding competition and inhibitory effects of higher concentrations of the solvent limit the complexity of the peptide libraries used in ELISpot assays [24], and despite the use of different fluorochromes [25, 26], only a small number of pMHC multimers can be used simultaneously for measuring T-cell responses. Given the functional heterogeneity and remarkable breadth of the EBV-specific CD4+ and CD8+ T-cell responses [21, 27, 28], as well as the often very small-sized blood samples obtained especially from very young IM patients, these limitations hamper comprehensive analyses of cell-mediated anti-viral responses.

Here, we sought to develop a novel multicolor flow cytometry-based assay that facilitates future investigations of immunopathological signatures potentially associated with acutely complicated and/or protracted IM. To expand the antigenic spectrum and to allow for simultaneous assessment of complex T-cell responses, various antigen preparations and combinations were tested, including EBV T-activated^®^ proteins [29], peptide pools covering the majority of immunodominant CD8+ T-cell epitopes [28], as well as EBV-like particles (EB-VLP) [30]. The latter cover the full spectrum of EBV structural antigens that have been previously identified as dominant targets of CD4+ T-cell responses in healthy EBV carriers and patients with IM [31–34].

## Methods

### Patients

Twenty patients (12 female, 8 male) with a median age of 9.5 (range 5 – 17) years were recruited into the Munich infectious mononucleosis (IMMUC) study, a large prospective, multicenter observational clinical study with the aim of defining biomarkers for acutely complicated and protracted disease (Table 1). Patients were diagnosed with symptomatic EBV primary infection by clinical and laboratory criteria and seen twice, for a first visit (v1) within 28 days (within two weeks for the sub-cohort of 20 patients of this work) after IM symptom onset (T_onset_), and for a follow-up visit (v2) usually within four to eight weeks after T_onset_. Blood samples and clinical data were collected at each visit.

**Table 1:** Study set-up

**Patient characteristics (*n* = 20)**	
**Age**	median 9.5 (range 5-17) years
**Female**	*n* = 12 (60.0%)
**Male**	*n* = 8 (40.0%)
**Study visits (v1, v2) after T**_**onset**_	
** Δ T**_**onset**_** to v1**	median 9.5 (range 2-14) days
** Δ T**_**onset**_** to v2**	median 29.0 (range 23-39) days
** Δ v1 to v2**	median 20.5 (range 11-33) days
**Diagnosis of IM**	

Initial manifestation of at least 1 of 4 typical clinical IM symptoms (tonsillopharyngitis, fever, lymphadenopathy, fatigue) and virological findings indicating recent EBV primary infection (ELISA, Immunoblot, PCR)

Primary infection with EBV was confirmed by combined results of EBV immunoassay, i.e. the Architect^®^ EBV panel (EBNA-1 IgG, VCA IgG, -IgM, analyzed on an Architect i1000SR, Abbott) and an EBV Immunoblot (recomLINE EBV IgG/IgM, Mikrogen), and by EBV-specific PCR as described [35]. An overview of the viral loads is summarized in Table 2.

**Table 2:** EBV load

**ID**	**Days after T**_**onset**_ **[visit 1]**	**Viral load** **[Geq/10**^**5**^** PBMC]**	**Days after T**_**onset**_ **[visit 2]**	**Viral load** **[Geq/10**^**5**^** PBMC]**
**1**	10	722.0	34	8.0
**2**	5	331.0	31	47.3
**3**	14	93.0	29	86.0
**4**	9	339.0	31	22.1
**5**	7	<LOQ	29	26.7
**6**	12	32.0	37	27.5
**7**	6	122.0	26	11.3
**8**	2	10.9	30	<LOQ
**9**	13	16.0	26	65.0
**10**	13	10.0	26	n.d.
**11**	6	118.0	36	0.0
**12**	10	88.6	31	0.0
**13**	11	11.6	38	28.0
**14**	10	525.0	27	n.d.
**15**	6	2,180.0	39	8.8
**16**	8	58.8	24	21.0
**17**	12	n.d.	23	<LOQ.
**18**	8	139.0	28	n.d.
**19**	10	124.0	27	9.0
**20**	9	107.0	29	26.0

*Geq* Genome equivalents, *PBMC* Peripheral blood mononuclear cells, *<LOQ* below the limit of quantification, *n.d*. not determined

### Production, purification, and quantification of EB-VLP

EB-VLP were harvested from supernatants of the producer cell line 293/TR- transiently transfected with a BZLF1 expression plasmid as described previously [36]. Likewise, wildtype (wt)-EBV was harvested from supernatant of the producer cell line 293/2089 after induction of the lytic cycle [37]. Both adherent cell lines were cultivated in RPMI1640 medium supplemented with 10% FCS, 1 mM sodium pyruvate, 2 mM L-glutamine, 1% non-essential amino acids, 50 µg/mL gentamycin, and 100 µg/mL hygromycin B (Thermo Fisher Scientific).

Supernatants containing wt-EBV or EB-VLP were sterile-filtered (0.45 µm Millex-HA) and concentrated by ultracentrifugation (2 h at 85000 x g). The pellets were resuspended in PBS and aliquots stored at -80 °C. The titer of wt-EBV was determined by measuring genome equivalents (Geq) by quantitative real-time PCR using primers specific for the viral BALF5 gene and was found to be 1.3x10^6^ Geq/mL [32].

### Flow cytometry-based quantification of EB-VLP

The concentration of EB-VLP was assessed by flow cytometry using wt-EBV as reference. 1 x 10^5^ Elijah cells were incubated with different volumes of EB-VLP or wt-EBV supernatant for 16 h on ice. Cells were then washed in ice-cold FACS buffer (PBS with 1% bovine serum albumin and 0.05% NaN_3_), incubated for 30 min with a purified mouse monoclonal antibody directed against gp350 (clone 72A1), washed twice in ice-cold FACS buffer, incubated for 30 min with a secondary Cy-5- or PE-conjugated goat-anti-mouse antibody (GE Health Care, Amersham, dilution 1:200), washed twice and then resuspended in 500 µL ice-cold FACS buffer containing 0.5 mg/mL propidium iodide. Subsequently, cells were analyzed in a BD FACScan using CellQuest software (Additional file 1: Fig. S1).

### T-cell recognition assays

For assessing antigenicity of EB-VLP, T-cell recognition assays were performed as described [33]. Briefly, increasing amounts of wt-EBV or EB-VLP were pulsed overnight on 1 x 10^5^ cells of an autologous lymphoblastoid cell line (LCL) that had been established by infection with a viral mutant lacking BZLF1 and, thus, incapable of expressing lytic cycle proteins. Next day, 1 x 10^5^ cells of the CD4+ T-cell clones gp1D6 or JM-N-1H7, specific for epitopes derived from gp350 and BNRF1, respectively, were added per well for 16 h [32, 33]. Subsequently, IFN-γ concentration in the supernatant was quantified by enzyme-linked immunosorbent assay according to the manufacturer’s instructions (Mabtech) (Additional file 2: Fig. S2).

### Isolation and cryopreservation of peripheral blood mononuclear cells

Human peripheral blood mononuclear cells (PBMC) were isolated within 4 h after collection of EDTA-anticoagulated whole blood by Ficoll (PAN-Biotech) density gradient centrifugation as described previously [38]. PBMC were washed twice using RPMI-10 (RPMI1640 medium supplemented with 1% Penicillin/Streptomycin and 10% heat inactivated fetal calf serum) and counted by an automated cell counter (ViCell XR). The median PBMC number obtained per mL whole blood was 2.9 x 10^6^ PBMC with a median viability of 98.2%. PBMC were frozen at a concentration of 1 x 10^7^ PBMC per 1 mL freezing medium (FCS supplemented with 10% DMSO) in 1.8 mL cryotubes (Thermo Fisher Scientific), using a freezing container (Mr. Frosty) and stored overnight at -80 °C. For long-term storage, PBMC were transferred into the vapor phase of a liquid N_2_ tank. PBMC were thawed in a 37 °C water bath, rested overnight, and counted using an ImmunoSpot Ultimate UV Image analyzer (CTL Europe GmbH) as described previously [39, 40].

### Quantification of EBV-reactive T cells by intracellular cytokine staining

EB-VLP [28], T-activated^®^ EBV-derived BZLF1 and EBNA3A recombinant proteins (Lophius Biosciences), and an EBV B95.8 strain-derived synthetic peptide pool (PP) (JPT Peptide Technologies, purity > 90%) comprising 94 published T-cell epitopes [41] were tested as recall antigens. Each peptide in the pool had a concentration of 100 µg/mL (Additional file 3: Table S3). For T-cell stimulation, 1 x 10^6^ PBMC in 100 µL RPMI-10 media supplemented with 1 µg/mL of the agonistic anti-CD28 antibody (clone L293, BD Biosciences) were brought out per well of a 96-well polypropylene U-bottom microtiter plate (BD Falcon). PBMC of each subject were incubated with 50 µL/mL EB-VLP (equivalent to 6.5 x 10^5^ copies of wt-EBV), 10 µg/mL T-activated^®^ EBV-BZLF1 and EBV-EBNA3A proteins (TP), and 2.5 µg/mL EBV-PP. For each sample, a mock-stimulated sample was run in parallel to define background activity. After 1 h stimulation with EBV-PP and after 3 h of stimulation with EB-VLP and TP at 37 °C in 5% CO_2_, 10 μg/mL of Brefeldin A (Sigma-Aldrich) was added to the cell suspension for 4 h at 37 °C in 5% CO_2_. Next, PBMC were labelled with the LIVE/DEAD™ Fixable Blue Dead Cell Stain Kit (Thermo Fisher Scientific) in a total volume of 100 μL for 30 min at 4 °C in the dark and washed twice with 200 µL FACS buffer (BD Biosciences). After centrifugation (560 x g, 4 °C, 5 min), PBMC were fixed for 20 min at 4 °C in the dark in 100 µL of an intracellular fixation buffer (Intracellular Fixation Buffer, Thermo Fisher Scientific). After two wash steps with 200 µL/well Perm/Wash solution (Cytofix/Cytoperm Kit; BD Biosciences) and a centrifugation step (710 x g, 4 °C, 5 min), PBMC were stained with mouse anti-human CD28 (1.0 µg/mL, BD Biosciences) and antibodies depicted in Additional file 4: Table S4 in a total volume of 80 µL Perm/Wash buffer including a brilliant violet buffer (BD Pharmingen Stain Buffer, BD Biosciences) for 30 min at 4 °C in the dark. Single color compensation was performed using 25 µL of compensation beads (UltraComp eBeads and ArC™ Amine Reactive Compensation Bead Kit for LIVE/DEAD compensation, both from Thermo Fisher Scientific) following the instructions of the manufacturer. Cells and beads were washed twice and finally resuspended in 300 µL FACS buffer and stored cold and in the dark until analysis.

Data acquisition and calculation of positive responses was performed as described previously [39]. The gating strategy for flow cytometric analysis of *ex vivo* stimulated PBMC is shown as Additional file 5: Fig. S5. Each gate was set in the negative control sample and then adjusted to peptide-stimulated cells with consideration of T-cell receptor downregulation. Two independent audits by different individuals were performed to control the gating. According to the differential expression of CD4 and CD8 proteins, T-cell subpopulations were defined.

### Statistical analysis

All results were included in the analysis and no outliers were excluded. All tests were two-sided and conducted on exploratory 5% significance levels. Effect measures are presented with 95% confidence intervals. Nonparametric statistical tests were applied in all cases. Mann-Whitney-Test was used for comparing different groups. All statistical tests were performed using GraphPad Prism Software (Version 7, GraphPad Software).

## Results

Flow cytometry-based intracellular cytokine staining was used to assess frequency, specificity, and function of EBV-reactive CD4+ and CD8+ T cells *ex vivo* in the peripheral blood of individuals at two time points (v1 and v2; median 9.5 and 29 days after symptom onset (T_onset_), respectively) within the early phase of IM. Three different formulations were used as recall antigens and comparatively analyzed; (i) T-activated EBV proteins BZLF1 and EBNA3A (hereinafter referred to as BZLF1 and EBNA3A) that can access both MHC class I and II antigen processing and presentation pathways [29, 42], (ii) EB-VLP covering structural antigens and enabling monitoring of CD4+ T-cell responses against late lytic cycle antigens [43, 44] and (iii) an EBV-derived peptide pool covering 94 well-characterized CD8+ T-cell epitopes from latent, immediate early, and early lytic cycle proteins [41, 45].

### High frequencies of EBV-derived PP-specific CD8+ T-cell responses in the early phase of IM

Following stimulation with the different antigen formulations, the magnitude of the virus-specific CD4+ T-cell responses against EBNA3A, BZLF1, and EB-VLP, and the CD8+ T-cell responses against EBNA3A, BZLF1, EB-VLP, and PP was assessed by intracellular staining of IFN-γ, IL-2, and TNF. Cytokine responses were detected in both CD4+ and CD8+ T-cell subsets in ≥95% of the patient samples. With 75% (15/20 patients), the lowest response rates were detected in CD8+ T cells stimulated with EB-VLP at v2 (Additional file 6: Table S6).

**Fig. 1:**
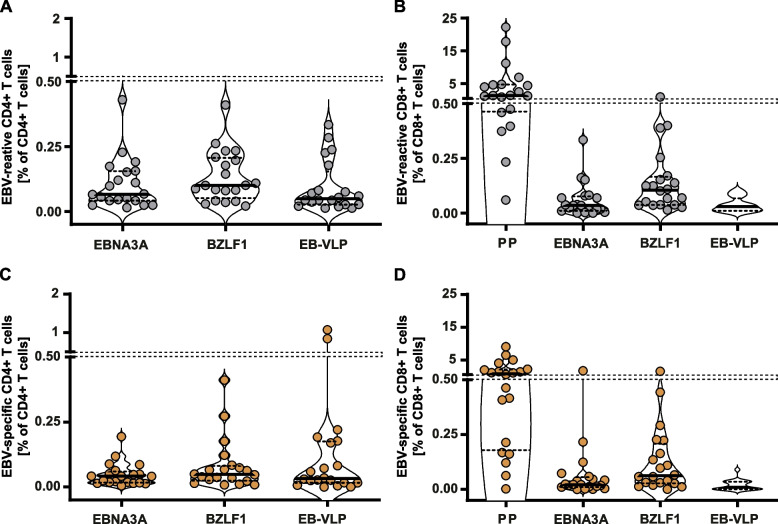
Total cytokine responses of EBV-reactive CD4+ and CD8+ T cells. Depicted are the frequencies of EBV-reactive CD4+ and CD8+ T cells upon stimulation of PBMC with T-activated EBNA3A and BZLF1 proteins, EBV-like particles** (**EB-VLP), and a synthetic EBV-derived peptide pool (PP) at visit 1 (v1) (**A, B**) (grey dots) and visit 2 (v2) (**C, D**) (brown dots). Total cytokine responses were calculated by adding together the frequencies of EBV-reactive IFN-γ, IL-2, and/or TNF-positive T cells within the CD4+ or CD8+ T-cell populations. Dots represent the patient samples analyzed (*n* = 20), the horizontal line marks the median, dashed lines the 25^th^ and 75^th^ percentiles. For reasons of clarity, the x-axis was moved to -0.05. v1: 2-14 days after symptom onset; v2: 23-39 days after symptom onset

At v1, the percentage of BZLF1-reactive CD4+ T cells was slightly higher (median 0.10%) than those directed against EBNA3A and EB-VLP (median 0.07% and 0.05%, respectively) (Fig. 1A). At v2, the percentage of reactive CD4+ T cells had declined to median frequencies of 0.05, 0.04, and 0.03% following BZLF1, EBNA3A, and EB-VLP stimulation, respectively (Fig. 1C).

The percentage of PP-reactive CD8+ T cells at v1 was significantly higher (median 1.39%) than those recognizing BZLF1 or EBNA3A (median 0.11% and 0.04%) *(p* < 0.0001) (Fig. 1B). Although low in numbers, EB-VLP-reactive CD8+ T cells were present in all patients (median 0.03%) (Fig. 1B). As observed for CD4+ T cells, the magnitude of virus-specific CD8+ T-cell response was generally lower at v2, especially after PP (median 0.8%), and EB-VLP (median 0.01%) (*p* = 0.001 and *p* = 0.009, respectively) stimulation (Fig. 1D).

**Fig. 2:**
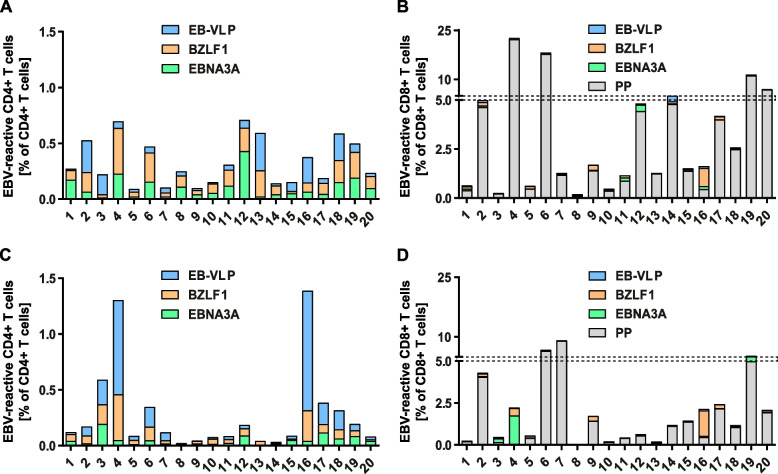
Individual EBV-specific CD4+ and CD8+ T-cell responses at visits 1 and 2. Stacked bars depict the frequencies of EBV-reactive CD4+ and CD8+ T cells upon stimulation of PBMC from 20 IM patients with T-activated EBNA3A and BZLF1 proteins, EBV-like particles** (**EB-VLP), and a synthetic EBV-derived peptide pool (PP) at visit 1 (v1) (**A, B**) and visit 2 (v2) (**C, D**). Total cytokine responses were calculated by adding together the frequencies of EBV-reactive IFN-γ, IL-2, and/or TNF-positive T cells within the CD4+ or CD8+ T-cell population. v1: 2-14 days after symptom onset; v2: 23-39 days after symptom onset

Among individual IM patients, magnitude, dynamics, and specificity of CD4+ and CD8+ T-cell responses varied greatly (Fig. 2). The combined CD4+ T-cell responses against all recall antigens strongly differed between patients at v1 (range 0.09-0.71%) and diverged further over time to 0.02-1.39% at v2. In addition, the overall decrease in virus-specific CD4+ T-cell responses from v1 to v2 was mostly caused by a decline in CD4+ T cells targeting EBNA3A, while responses against BZLF1 were maintained and EB-VLP-reactive CD4+ T cells decreased in most, but sharply increased in two patients (Fig. 2A, C).

Similar intra- and inter-individual variations in magnitude and specificity of the virus-specific immune response were also observed for CD8+ T cells. Due to the high frequency of PP-reactive T cells in some donors, the range of total responses was even broader (0.2-22.69% at v1 and 0.004-9.13% at v2) and the decline in the magnitude of the response from v1 to v2 was mostly caused by a contraction of the PP-specific CD8+ T-cell population (Fig. 2B, D). Changes in antigen specificity were occurring but were less pronounced than within CD4+ T cells (e.g., patients #3 and #4) (Fig. 2B, D). Except for the general decrease over time, the dynamic changes in individual CD4+ and CD8+ T-cell responses were patient-specific and did not correlate with the time from disease onset (T_onset_) (data not shown).

In summary, EBV-derived PP-specific CD8+ T-cell response strongly dominated in the early phase of IM. However, all three EBV-derived antigen formulations elicited measurable recall responses in all patients and allowed for a more comprehensive characterization of the EBV-specific T-cell response during the course of IM *ex vivo*.

### Functional properties of EBV-reactive CD4+ and CD8+ T cells

Poly-functional T cells (PFC) produce multiple effector molecules upon stimulation and have been associated with more effective control of persistent viral infections [21, 46]. To gain insight into the functionality of EBV-reactive T cells, secretion of IFN-γ, TNF, and IL-2 by CD4+ and CD8+ T cells was analyzed.

**Fig. 3:**
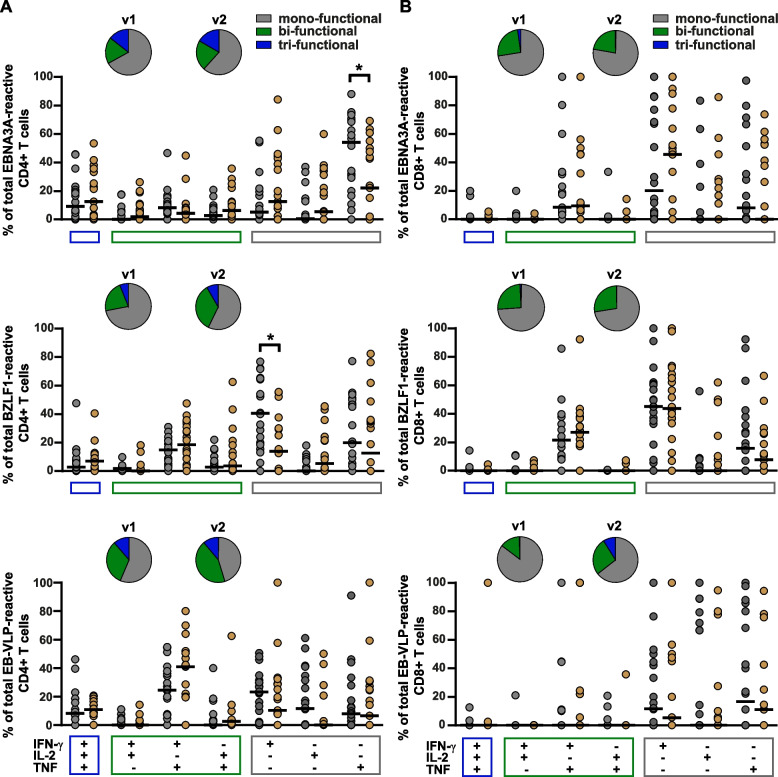
Functionality of EBV-reactive CD4+ and CD8+ T cells at visit 1 and 2. The colored dots and black lines depict individual frequencies and median frequencies, respectively, of antigen-reactive CD4+ (A) or CD8+ (B) T cells characterized by the indicated combination of cytokines (IFN-γ, IL-2, TNF) at visit 1 (v1) (grey dots) and visit 2 (v2) (brown dots) in samples from 20 patients each. The depicted pies show the average proportion of antigen-reactive mono- (cells producing only one of the cytokines), bi- (cells producing two of the respective cytokines), and tri-functional (cells producing all three cytokines simultaneously) CD4+ (**A**) and CD8+ (**B**) T cells at visits 1 and 2. v1: 2-14 days after symptom onset; v2: 23-39 days after symptom onset. * *p* < 0.05

Irrespective of the recall antigen, virus-specific CD4+ T cells at v1 were mainly mono-functional (mean 64.92%, range 56.15-71.71%) with minor proportions of cells producing two or three cytokines (Fig. 3A). The main cytokine produced by mono-functional T cells was either IFN-γ or TNF, and both together were the main cytokines produced by bi-functional CD4+ T cells (Fig. 3A). A similar phenotype with a slight trend towards multi-functional CD4+ T cells was detected at v2. While the percentage of IFN-γ^+^ or TNF^+^ mono-functional CD4+ T cells was reduced (mean 53.83%, range 43.01-61.43%), the proportion of bi-functional, mainly Th1 polarized IFN-γ^+^/TNF^+^ CD4+ T cell subpopulations had increased (v1: mean 24.25%, range 18.45-32.21%, v2: mean 32.72%, range 21.90-41.71%) (Fig. 3A). Although in individual patients up to 50% of all EBNA3A- or EB-VLP-reactive CD4+ T cells were IFN-γ^+^/TNF^+^/IL2^+^, PFC appeared only rarely in the majority of patients during the early phase after T_onset_ (v1 vs. v2: mean 10.83-11.96%) (Fig. 3A).

**Fig. 4:**
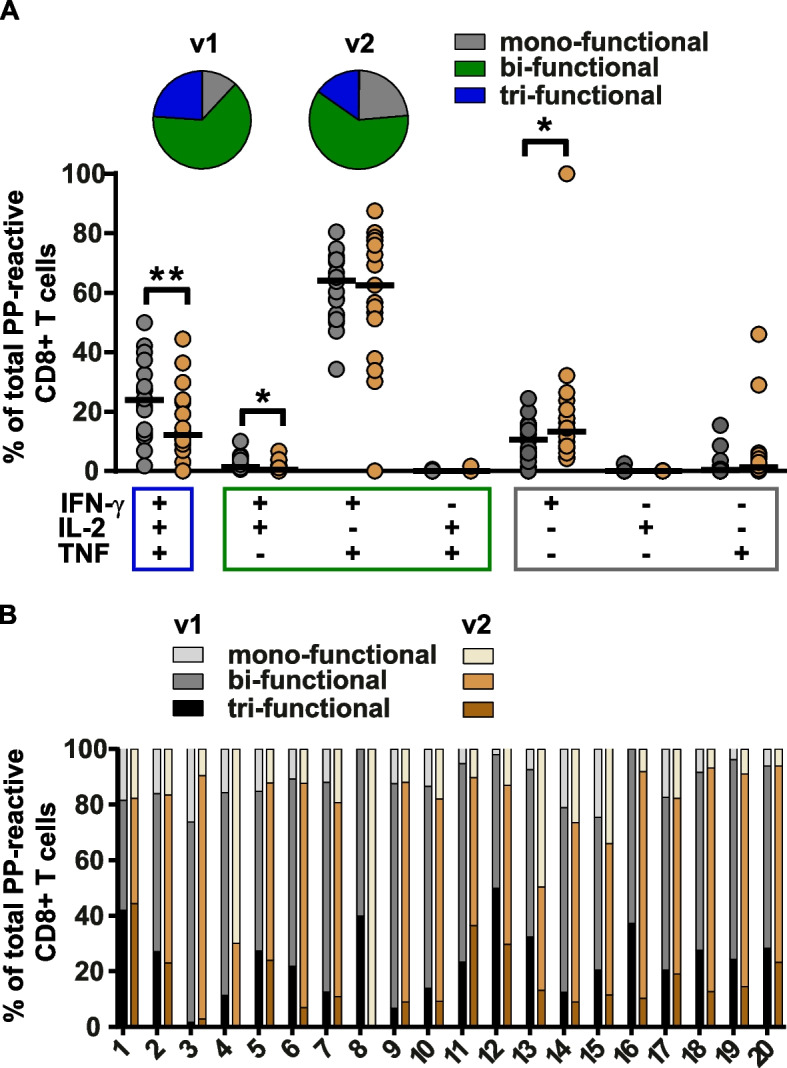
Functionality of PP-reactive CD8+ T cells at visit 1 and 2. (**A**) Frequencies of PP-reactive CD8+ T cells in 20 IM patients at visit 1 (v1, grey dots) and visit 2 (v2, brown dots) are depicted for each functional combination of the three cytokines (IFN-γ, IL-2, TNF) analyzed. The colored dots and black lines represent individual frequencies and median frequencies of antigen reactive CD8+ T cells, respectively. The depicted pies show the average proportion of antigen-reactive mono- (cells producing only one of the cytokines), bi- (cells producing two of the respective cytokines), and tri-functional (cells producing all three cytokines simultaneously) CD8+ T cells at visits 1 and 2. * *p* < 0.05, ** *p* < 0.01. **B** Individual proportion of mono-, bi-, and tri-functional PP-reactive CD8+ T cells in the 20 IM patients at v1 and v2

At v1, CD8+ T cells recognizing BZLF1, EBNA3A, or EB-VLP were mainly mono-functional (mean 74.66%, range 65.14-85.22%) and most of the remaining cells were bi-functional, again with IFN-γ and TNF being the prevailing cytokines (mean 20.75%) (Fig. 3 B). This phenotypic pattern appeared to be stable, and the cytokine expression pattern barely changed from v1 to v2 (Fig. 3 B). By contrast, PP-reactive CD8+ T cells were mostly bi-functional (>60% at v1 and v2), produced IFN-γ and TNF, and had more tri-functional than mono-functional components (Fig. 4). Furthermore, the phenotype of PP-reactive CD8+ T cells was much less divers with IFN-γ^+^/TNF^+^ > IFN-γ^+^/TNF^+^/IL2^+^ > IFN-γ^+^, CD8+ T cells being the dominant T-cell subpopulations. While bi-functional T cells remained the largest population, the proportion of multi-functional T cells gradually declined from v1 to v2 (v1: 24.12%, v2: 15.55%).

Conversely, the proportion of mono-functional T cells increased from 11.79 to 23.42% (Fig. 4A). To examine whether this overall decline in multi-functionality was a general phenomenon or caused by disproportionate changes in few patients, as observed for specificity and magnitude, individual responses were analyzed (Fig. 4B). These experiments verified a consistent decline in multi-functional T cells in all patients and hence identified functionality as a possible marker for the acute phase of EBV infection.

These results demonstrated a pronounced multi-functional PP-reactive CD8+ T-cell response during the early phase of IM, whereas BZLF1-, EBNA3A-, and EB-VLP-reactive CD4+ and CD8+ T-cell responses were mainly mono-functional.

### Correlation analysis between EBV-specific T-cell responses and viral load

To analyze the relationship between viral load and EBV-specific CD4+ and CD8+ T-cell responses, EBV load was determined at v1 and v2 using real-time PCR. EBV load was highest at the time of the first visit (v1: median 112 Geq/10^5^ PBMC, range 10-2,180 Geq/10^5^ PBMC) and then declined rapidly (v2: median 22 Geq/10^5^ PBMC, range 0-47 Geq/10^5^ PBMC) (Table 2).

At v1, a moderate correlation between viral load and EBNA3A, EB-VLP, and PP-reactive CD8+ T cells was noted (*r*_*s*_ = 0.345, 0.418, and 0.356, with p = n.s., respectively) (Table 3). At v2, viral load moderately correlated with BZLF1 and EB-VLP-reactive CD8+ T cells (*r*_*s*_ = 0.462 and 0.349, with p = n.s., respectively), but no longer with EBNA3A or PP-reactive CD8+ T cells (Additional file 7: Table S7).

## Discussion

The clinical picture of IM is considered to be a correlate of aberrant immune responses to primary EBV infection, and IM is associated with an increased risk for developing distinct EBV-associated diseases later in life. Hence, understanding the differences in EBV-specific immune responses of individuals with primary EBV infection may identify signatures of effective antiviral immunity as well as risk parameters for acute or late IM complications sequelae and other EBV-associated diseases. Given the broadness and functional heterogeneity of the EBV-specific T-cell responses, immune profiling assays should include a maximum set of EBV antigens and enable functional characterization of the responding cell population, ideally in small-sized samples as obtained from pediatric IM patients. Here, we tested the ability of three antigen formulations to reactivate virus-specific T cells *ex vivo* and to facilitate their functional characterization.

Because the number of known EBV epitopes presented on MHC class II is still low, full-length proteins that are processed endogenously were used as sources of antigen for assessing CD4+ T-cell responses. EB-VLP contain about half of the more than 80 viral proteins that are encoded on the viral genome [47]. Following receptor-mediated uptake and presentation on MHC II, peptides derived from virion proteins have been shown to efficiently reactivate a broad set of T-cell specificities in PBMC of healthy donors [5, 32, 33, 43, 48–50], and even of immunosuppressed patients [44]. EB-VLP-specific CD4+ T-cell responses were detected in all, except in one patient at time point v2, when responses were generally lower than at the time of diagnosis. Responses against EB-VLP were lower than those against EBNA3A and BZLF1 proteins, an unexpected finding considering the immunodominance of structural antigens in healthy virus carriers [43, 50]. These results may indicate that responses against EB-VLP develop later during the course of EBV infection. In fact, EB-VLP-specific responses strongly increased from v1 to v2 in two donors, whereas average and individual responses against EBNA3A generally declined during this time period. However, more time points during IM and subsequent convalescence need to be analyzed to verify such longitudinal changes in EB-VLP specificity. Moreover, including an age-matched healthy control group in future studies would allow for a comparing of EBV-specific immune responses during the acute phase and the healthy virus carrier state in children. However, such analyses are difficult to conduct because obtaining sufficient amounts of blood from healthy children for control purposes is ethically difficult to justify, and leftover blood samples from children are usually very limited in size, thereby precluding ex vivo analyses of T-cell responses without unwanted prior in vitro expansion.

CD4+ T-cell responses against all three antigen formulations comprised similar fractions of mono-, bi-, and multi-functional T cells, suggesting that the EBV-specific CD4+ T-cell response is functionally homogeneous. This is in line with *ex vivo* HLA class II tetramer analyses reporting a Th1-like phenotype in EBV-specific CD4+ T cells [5, 50, 51]. Extending these analyses to additional marker proteins, such as lineage-defining transcription factors or subtype-specific cytokines, may allow for subdivision into T-helper cell subsets and thereby provide further insight into the cellular composition and complexity of the response.

Low-frequent responses against EB-VLP were also detected in CD8+ T cells, which was unexpected because EB-VLP are not known to access the MHC class I antigen presentation pathway in PBMC. Likewise, T-activated proteins elicited CD8+ T-cell responses of similar magnitude *via* a still elusive presentation pathway [29]. Additional experiments are needed to determine whether the responding populations consist of “classical” CD8+ effectors, or less well-defined T-cell subsets [52, 53]. The latter possibility is supported by a similar functional phenotype of the responding T-cell populations, consisting mostly of mono-functional and a smaller proportion of bi-functional effectors. By contrast, PP-stimulated CD8+ T cells had significantly higher bi- and tri-functional components, suggesting that these cells belong to a different effector subtype. Responses against the peptide pool exceeded those against other antigen formulations by at least one order of magnitude, probably due to the massive expansion of these CD8+ T-cell effectors that is often observed during acute infection. These T cells typically recognize epitopes derived from immediate early and early lytic cycle proteins, and single T-cell specificities may account for several percent of the peripheral CD8+ T-cell compartment [5, 45]. As with other antigen formulations, individual patients displayed highly variable frequencies of responding CD8+ T cells, for which several potential reasons may exist. First, the epitopes included in the peptide pool are mostly presented on HLA class I alleles frequently found in the Caucasian population, but individual patients may express varying numbers of these restriction elements and, consequently, show varying responses. Second, in some patients, CD8+ T cells may have failed to respond to the B95.8-derived peptides in the pool because they were primed against polymorphic epitope variants expressed by the infecting viral strain [28, 41, 54]. Thus, including epitopes presented on less frequent HLA alleles as well as polymorphic epitope variants in the peptide pool may increase the number of responding CD8+ T cells in these patients. Alternatively, the virus-specific CD8+ T-cell response may inherently vary between individuals. This notion is supported by the highly variable T-cell responses observed against all different antigen formulations in this and in earlier studies. For example, LCL-stimulated T-cell preparations from different healthy donors were shown to vary in the CD4+ to CD8+ ratio from 98:2 to 2:98 [55], suggesting that the response in healthy donors can be dominated by different T-cell subsets. Also, opposite dynamics in the epitope-specific CD8+ T-cell response against latent and lytic cycle antigens were observed from primary to persistent phases of infection in adults [5, 56] as well as in children with symptomatic or asymptomatic primary infection [21]. Moreover, CD4+ T cells recognizing EBNA1 only develop several months after acute infection [57], while CD8+ T-cell responses against late antigens appear to increase in the long term [58]. Concomitant with these changes in antigen specificity, diverse poly-functional subtypes have been reported to emerge with time from primary infection, affecting cytokine production of CD4+ and CT8+ T cells [21] in the case of CD4+ T cells, also cytotoxicity cytotoxicity seems to play an important role [51, 59–61]. Our experiments add to these findings by showing that such shifts in T-cell specificity, magnitude, and functionality can already occur within 3 weeks, as observed from v1 to v2. Although merely intended as a pilot study with limited sample size, the correlation of viral titers at v1 and v2 with CD8+ T-cell responses against mostly different antigen formulations indicate that signatures of protective immunity may evolve with the immune response during IM. Together with the remarkable broadness of the EBV-specific T-cell response, these dynamic changes imply that the elucidation of protective antiviral responses depends on the utmost coverage of the EBV proteome in immunoassays. Yet, enlarging the antigenic spectrum might necessitate large-sized clinical samples, unless extra recall antigens are incorporated in existing antigen formulations. This, however, would come at the cost of losing information on antigen specificity, which might be a crucial determinant of immune signatures. First results from the analyses of 200 IM patients enrolled in the IMMUC study indicate that the immune monitoring approach described here can inform on still elusive T-cell response patterns associated with severe and complex IM disease and might ultimately facilitate the development of targeted immunotherapeutic strategies to restore protective antiviral immunity in patients at risk. In addition, a better understanding of the immunologic events during IM may also aid in the design of effective EBV vaccines in the future [62].

## Conclusions

The EBV-derived recall antigens were tested to be suitable for monitoring EBV-specific T-cell responses *ex vivo*. They facilitate thorough analyses of EBV-specific T-cell immunity and may support the identification of functional T-cell signatures that are linked to the course of disease.

### Supplementary Information

Additional file 1: Figure S1. Semi-quantification of EB-VLP using anti-gp350 flow cytometry. Elijah cells were incubated over night with different amounts of the wt-EBV (A) or EB-VLP (B) preparations. Next day, bound viral particles were quantified using gp350-specific flow cytometry. Based on the obtained fluorescence intensity signals, wt-EBV and EB-VLP concentrations were adjusted to yield similar MFI (C).

Additional file 2: Figure S2. Recognition of EB-VLP and wt-EBV by virion antigen-specific CD4+ T cells. CD4+ T cell clones specific for gp350 (A: GP1D6) and BNRF1 (B: JMN1H7) were tested for recognition of lymphoblastoid cell lines (LCL) pulsed with different amounts of purified EB-VLP or wt-EBV. Recombinant gp350 and BNRF1 protein served as positive controls. IFN-γ secretion was measured by ELISA.

Additional file 3: Table S3. Composition of the EBV-derived peptide pool.

Additional file 4: Table S4. Antibodies used in FACS analysis.

Additional file 5: Figure S5. Representative gating strategy of EBV-reactive T cells. Lymphocytes were gated based on FSC vs. SSC pseudocolor plot (A). After exclusion of dead cells (B) and doublets (C), CD3-positive lymphocytes were gated (D). Next, the CD3-positive population was gated on the expression of CD4 (E) and CD8 (F). Subsequently, CD4+ and CD8+ T cells were analysed for the expression of the intracellular cytokines IFN-γ, TNF, and IL-2 (G and H, respectively).

Additional file 6: Table S6. Reactivity of antigen reactive CD4+ and CD8+ T cells in individual IM patients.

Additional file 7.

## Data Availability

The data used to support the findings of this study are included within the article. The raw data supporting the conclusions of this article will be made available by the corresponding author on reasonable request.

## Abbreviations

DMSO: Dimethylsulfoxide
EBV: Epstein-Barr virus
EBV-HLH: Hemophagocytic lymphohistiocytosis
EB-VLP: Epstein-Barr virus-like particles
FCS: Fetal calf serum
Geq: Genome equivalents
IM: Infectious mononucleosis
IMMUC: Munich infectious mononucleosis study
LCL: Lymphoblastoid cell line
ME/CFS: Myalgic encephalomyelitis/chronic fatigue syndrome
PBMC: Peripheral blood mononuclear cells
PBS: Phosphate-buffered saline
PEM: Post-exertional malaise
PFC: Poly-functional T cells
PID: Primary immunodeficiencies
pMHC: Peptide-major histocompatibilty complex
PP: EBV-derived peptide pool
WT: Wild type
