# Supplementary material for: Evaluation of novel Epstein-Barr virus-derived antigen formulations for monitoring virus-specific T cells in pediatric patients with infectious mononucleosis
Source: Virol J. 2024 Jun 14;21:139. doi: 10.1186/s12985-024-02411-0 (PMC11179387; doi:10.1186/s12985-024-02411-0)
Supplement: Supplementary file 1 — Additional file 1: Figure S1. Semi-quantification of EB-VLP using anti-gp350 flow cytometry. Elijah cells were incubated over night with different amounts of the wt-EBV (A) or EB-VLP (B) preparations. Next day, bound viral particles were quantified using gp350-specific flow cytometry. Based on the obtained fluorescence intensity signals, wt-EBV and EB-VLP concentrations were adjusted to yield similar MFI (C). [file 12985_2024_2411_MOESM1_ESM.pdf]

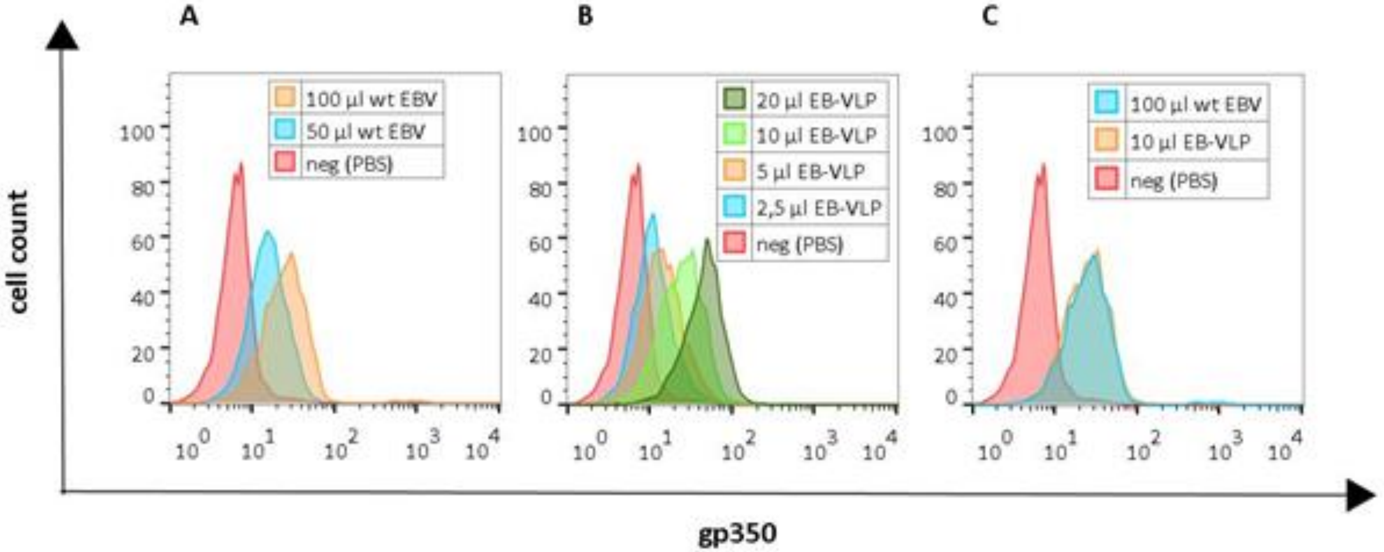


**Additional File 1 Fig. S1:** Semi-quantification of EB-VLP using anti-gp350 flow cytometry. Elijah cells were incubated over night with different amounts of the wt-EBV (A) or EB-VLP (B) preparations. Next day, bound viral particles were quantified using gp350-specific flow cytometry. Based on the obtained fluorescence intensity signals, wt-EBV and EB-VLP concentrations were adjusted to yield similar MFI (C).
