# Supplementary material for: Evaluation of novel Epstein-Barr virus-derived antigen formulations for monitoring virus-specific T cells in pediatric patients with infectious mononucleosis
Source: Virol J. 2024 Jun 14;21:139. doi: 10.1186/s12985-024-02411-0 (PMC11179387; doi:10.1186/s12985-024-02411-0)
Supplement: Supplementary file 2 — Additional file 2: Figure S2. Recognition of EB-VLP and wt-EBV by virion antigen-specific CD4+ T cells. CD4+ T cell clones specific for gp350 (A: GP1D6) and BNRF1 (B: JMN1H7) were tested for recognition of lymphoblastoid cell lines (LCL) pulsed with different amounts of purified EB-VLP or wt-EBV. Recombinant gp350 and BNRF1 protein served as positive controls. IFN-γ secretion was measured by ELISA. [file 12985_2024_2411_MOESM2_ESM.pdf]

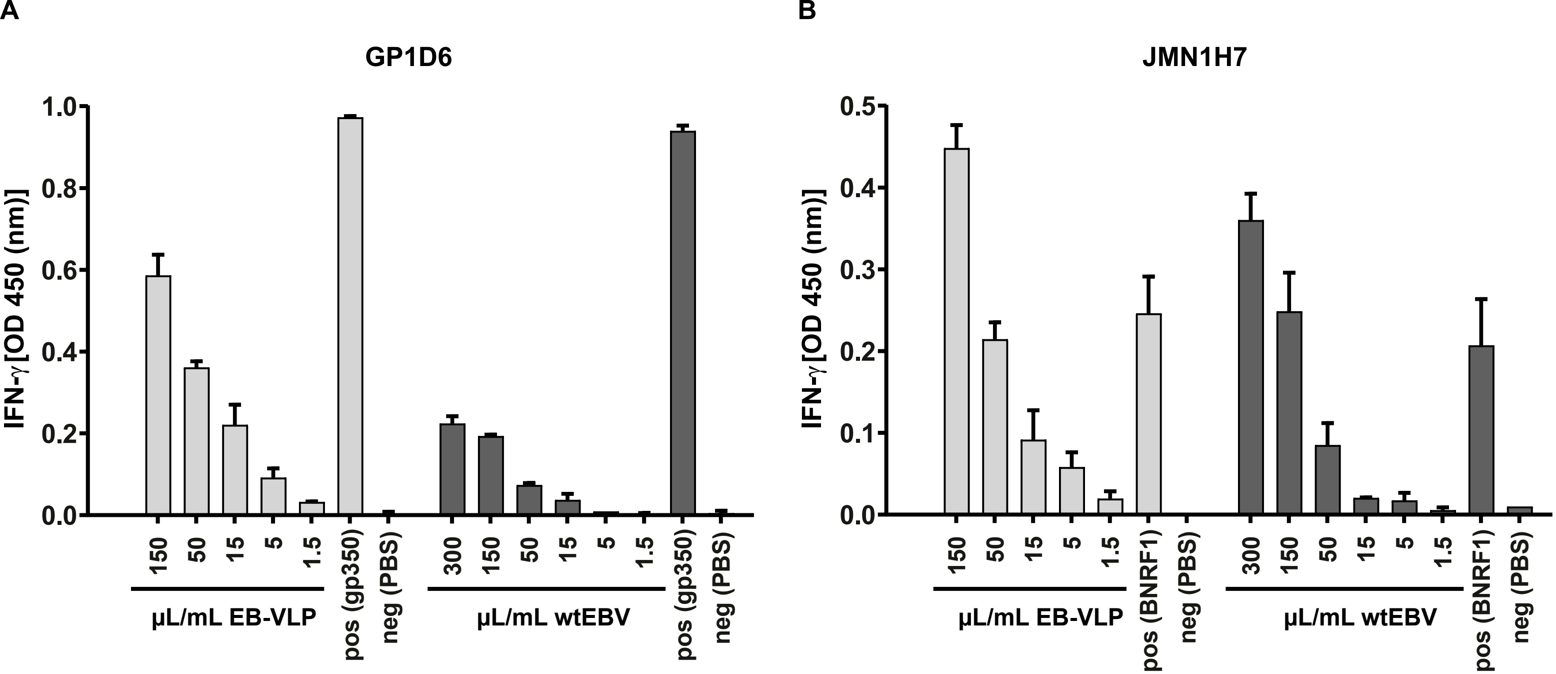

Additional File 2 Fig. S2: Recognition of EB-VLP and wt-EBV by virion antigen-specific CD4+ T cells. CD4+ T cell clones specific for gp350 (A: GP1D6) and BNRF1 (B: JMN1H7) were tested for recognition of lymphoblastoid cell lines (LCL) pulsed with different amounts of purified EB-VLP or wt-EBV. Recombinant gp350 and BNRF1 protein served as positive controls. IFN-γ secretion was measured by ELISA.
