# Supplementary material for: Evaluation of novel Epstein-Barr virus-derived antigen formulations for monitoring virus-specific T cells in pediatric patients with infectious mononucleosis
Source: Virol J. 2024 Jun 14;21:139. doi: 10.1186/s12985-024-02411-0 (PMC11179387; doi:10.1186/s12985-024-02411-0)
Supplement: Supplementary file 3 — Additional file 3: Table S3. Composition of the EBV-derived peptide pool. [file 12985_2024_2411_MOESM3_ESM.pdf]

Additional file 3: Table S3. Composition of the EBV-derived peptide pool.

#	Virus phase	Antigen	Amino acid†	Sequence‡	HLA class I restriction
**1**	Latent	EBNA1	72-80	**RPQKRPSCI**	B7
**2**	Latent	EBNA1	407-417	**HPVGEADYFEY**	B*35:01
**3**	Latent	EBNA1	508-517	**FVYGGSKTSL**	C03/04, A*68:02
**4**	Latent	EBNA1	518-526	**YNLRRGTAL**	B8
**5**	Latent	EBNA1	528-536	**IPQCRLTPL**	B7
**6**	Latent	EBNA1	566-574	**LQTHIFAEV**	A*02:06
**7**	Latent	EBNA1	574-582	**VLKDAIKDL**	A*02:03
**8**	Latent	EBNA2	14-23	**YHLIVDTDSL**	B38
**9**	Latent	EBNA2	42-51	**DTPLIPLTIF**	A2/B51
**10**	Latent	EBNA2	127-135	**LASAMRML**	B57/58
**11**	Latent	EBNA2	185-194	**QPRLTPPQPL**	B7
**12**	Latent	EBNA2	234-242	**RPTELQPTP**	B55
**13**	Latent	EBNA2	377-384	**TSSPSMPEL**	C*03:04
**14**	Latent	EBNA3A	158-166	**QAKWRLQTL**	B8
**15**	Latent	EBNA3A	176-184	**AYSSWMYSY**	B*30:02
**16**	Latent	EBNA3A	246-253	**RYSIFFDY**	A24
**17**	Latent	EBNA3A	325-333	**FLRGRAYGI**	B8
**18**	Latent	EBNA3A	378-387	**KRPPIFIRRL**	B27
**19**	Latent	EBNA3A	406-414	**LEKARGSTY**	B62
**20**	Latent	EBNA3A	450-458	**HLAAQGMAY**	B62
**21**	Latent	EBNA3A	458-466	**YPLHEQHGM**	B*35:01
**22**	Latent	EBNA3A	491-499	**VFSDGRVAC**	A29
**23**	Latent	EBNA3A	502-510	**VPAPAGPIV**	B7
**24**	Latent	EBNA3A	596-604	**SVRDRLARL**	A2
**25**	Latent	EBNA3A	603-611	**RLRAEAQVK**	A3
**26**	Latent	EBNA3A	617-625	**VQPPQLTQV**	B*46:01
**27**	Latent	EBNA3B	149-157	**HRCQAIRKK**	B*27:05
**28**	Latent	EBNA3B	217-225	**TYSAGIVQI**	B*24:02
**29**	Latent	EBNA3B	243-253	**RRARSLSAERY**	B*27:02
**30**	Latent	EBNA3B	279-287	**VSFIEFVGW**	B58
**31**	Latent	EBNA3B	399-408	**AVFDRKSDAK**	A11
**32**	Latent	EBNA3B	416-424	**IVTDFSVIK**	A11
**33**	Latent	EBNA3B	488-496	**AVLLHEESM**	B*35:01
**34**	Latent	EBNA3B	657-666	**VEITPYKPTW**	B44
**35**	Latent	EBNA3C	163-171	**EGGVGWRHW**	B*44:03
**36**	Latent	EBNA3C	213-222	**QNAARTLNTF**	B62
**37**	Latent	EBNA3C	249-258	**LRGKWQRRYR**	B*27:05
**38**	Latent	EBNA3C	258-266	**RRIYDLIEL**	B*27:02/04/05
**39**	Latent	EBNA3C	271-278	**HHIWQNLL**	B39
**40**	Latent	EBNA3C	281-293	**EENLLDFVRFMGV**	B*44:02, A*02:01
**41**	Latent	EBNA3C	335-343	**KEHVIQNAF**	B*44:02
**42**	Latent	EBNA3C	343-351	**FRKAQIQGL**	B*27:05
**43**	Latent	EBNA3C	881-889	**QPRAPIRPI**	B7
**44**	Latent	EBNA-LP	473-481	**SLREWLLRI**	A2
**45**	Latent	LMP1	38-46	**FWLYIVMSD**	n.d.
**46**	Latent	LMP1	51-60	**ALLVLYSFAL**	A2
**47**	Latent	LMP1	72-82	**FRRDLLCPLGA**	B40
**48**	Latent	LMP1	125-133	**YLLEMLWRL**	A2, A68, A69
**49**	Latent	LMP1	156-167	**IALYLQQNWWTL**	A2, B57, B58

**50**	Latent	LMP1	166-174	**TLLVDLLWL**	A2
**51**	Latent	LMP1	375-386	**DPHGPVQLSYYD**	B51
**52**	Latent	LMP2A	1-9	**MGSLEMVPM**	B*35:01
**53**	Latent	LMP2A	125-132	**LPVIVAPYL**	B53
**54**	Latent	LMP2A	131-139	**PYLFWLAAI**	A23
**55**	Latent	LMP2A	144-152	**FTASVSTVV**	A68
**56**	Latent	LMP2A	200-208	**IEDPPFNSL**	B*40:01
**57**	Latent	LMP2A	236-245	**RRRWRRLTVC**	B*14:01, B*27:04
**58**	Latent	LMP2A	240-251	**RRLTVCGGIMFL**	A1, B27
**59**	Latent	LMP2A	257-265	**LIVDAVLQL**	A*02:04/17
**60**	Latent	LMP2A	293-301	**GLGTLGAAL**	A2
**61**	Latent	LMP2A	329-337	**LLWTLVVLL**	A*02:01
**62**	Latent	LMP2A	340-352	**SSCSSCPLSKILL**	A11, B8
**63**	Latent	LMP2A	350-359	**ILLARLFLY**	A29
**64**	Latent	LMP2A	356-364	**FLYALALLL**	A2
**65**	Latent	LMP2A	419-427	**TYGPVFMCL**	A24
**66**	Latent	LMP2A	426-434	**CLGGLLTMV**	A*02:01
**67**	Latent	LMP2A	442-455	**VMSNTLLSAWILTA**	A2, A25
**68**	Latent	LMP2A	453-461	**LTAGFLIFL**	A*02:06
**69**	Lytic	BMLF1	244-252	**KDTWLDARM**	n.d.
**70**	Lytic	BMLF1	259-267	**GLCTLVAML**	A*02:01
**71**	Lytic	BMLF1	376-384	**DEVEFLGHY**	B18
**72**	Lytic	BMLF1	414-423	**SRLVRAILSP**	B14
**73**	Lytic	BMRF1	20-28	**CYDHAQTHL**	A24
**74**	Lytic	BMRF1	208-216	**TLDYKPLSV**	A*02:01
**75**	Lytic	BMRF1	268-276	**YRSGIIAVV**	B39, C6
**76**	Lytic	BMRF1	286-295	**LPLDLSVILF**	A11, B35, B53
**77**	Lytic	BRLF1	25-37	**LVSDYCNVLNKEF**	A*02:05, B18
**78**	Lytic	BRLF1	91-99	**AENAGNDAC**	B45
**79**	Lytic	BRLF1	109-117	**YVLDHLIVV**	A*02:01
**80**	Lytic	BRLF1	134-142	**ATIGTAMYK**	A11
**81**	Lytic	BZLF1	14-23	**TPDPYQVPFV**	B51
**82**	Lytic	BZLF1	16-26	**DPYQVPFVQAF**	B7
**83**	Lytic	BZLF1	44-52	**LPCVLWPVL**	B7
**84**	Lytic	BZLF1	52-64	**LPEPLPQGQLTAY**	B*35:01/08
**85**	Lytic	BZLF1	65-74	**HVSTAPTGSW**	A25
**86**	Lytic	BZLF1	66-75	**VSTAPTGSWF**	B*58:01
**87**	Lytic	BZLF1	77-89	**APQPAPENAYQAY**	B*35:01/08
**88**	Lytic	BZLF1	122-130	**VQTAAAVVF**	B*15:01, B58, B62
**89**	Lytic	BZLF1	169-183	**EECDSELEIKRYKNR**	B18, B*44:03
**90**	Lytic	BZLF1	179-187	**RYKNRVASR**	A31
**91**	Lytic	BZLF1	190-197	**RAKFKQLL**	B8
**92**	Lytic	BZLF1	192-200	**KFKQLLQHY**	A30
**93**	Lytic	BZLF1	197-205	**LQHYREVAA**	C8
**94**	Lytic	BZLF1	209-217	**SENDRLRLL**	B49, B60

†Protein sequence was derived from the B95.8 strain of EBV. ‡ In some cases, two partially overlapping epitopes were combined in one peptide (grey). n.d. = not determined.
