# Supplementary material for: Evaluation of novel Epstein-Barr virus-derived antigen formulations for monitoring virus-specific T cells in pediatric patients with infectious mononucleosis
Source: Virol J. 2024 Jun 14;21:139. doi: 10.1186/s12985-024-02411-0 (PMC11179387; doi:10.1186/s12985-024-02411-0)
Supplement: Supplementary file 4 — Additional file 4: Table S4. Antibodies used in FACS analysis. [file 12985_2024_2411_MOESM4_ESM.pdf]

Additional file 4: Table S4. Antibodies used in FACS analysis.

Antibody	Fluorochrome	Vendor	Clone	Assay concentration [µg/mL]
**CD3**	BV510	BioLegend	SK7	0.08
**CD4**	eF450	eBiosciences	SK3	0.04
**CD8**	ECD	Beckman Coulter	SFCI21Thy2D3	3.1
**IFN-γ**	Al700	BD Biosciences	B27	0.01
**TNF**	BV785	BioLegend	MAb11	0.5
**IL-2**	FITC	BD Biosciences	5344.111	0.3
