# Supplementary material for: Evaluation of novel Epstein-Barr virus-derived antigen formulations for monitoring virus-specific T cells in pediatric patients with infectious mononucleosis
Source: Virol J. 2024 Jun 14;21:139. doi: 10.1186/s12985-024-02411-0 (PMC11179387; doi:10.1186/s12985-024-02411-0)
Supplement: Supplementary file 5 — Additional file 5: Figure S5. Representative gating strategy of EBV-reactive T cells. Lymphocytes were gated based on FSC vs. SSC pseudocolor plot (A). After exclusion of dead cells (B) and doublets (C), CD3-positive lymphocytes were gated (D). Next, the CD3-positive population was gated on the expression of CD4 (E) and CD8 (F). Subsequently, CD4+ and CD8+ T cells were analysed for the expression of the intracellular cytokines IFN-γ, TNF, and IL-2 (G and H, respectively). [file 12985_2024_2411_MOESM5_ESM.pdf]

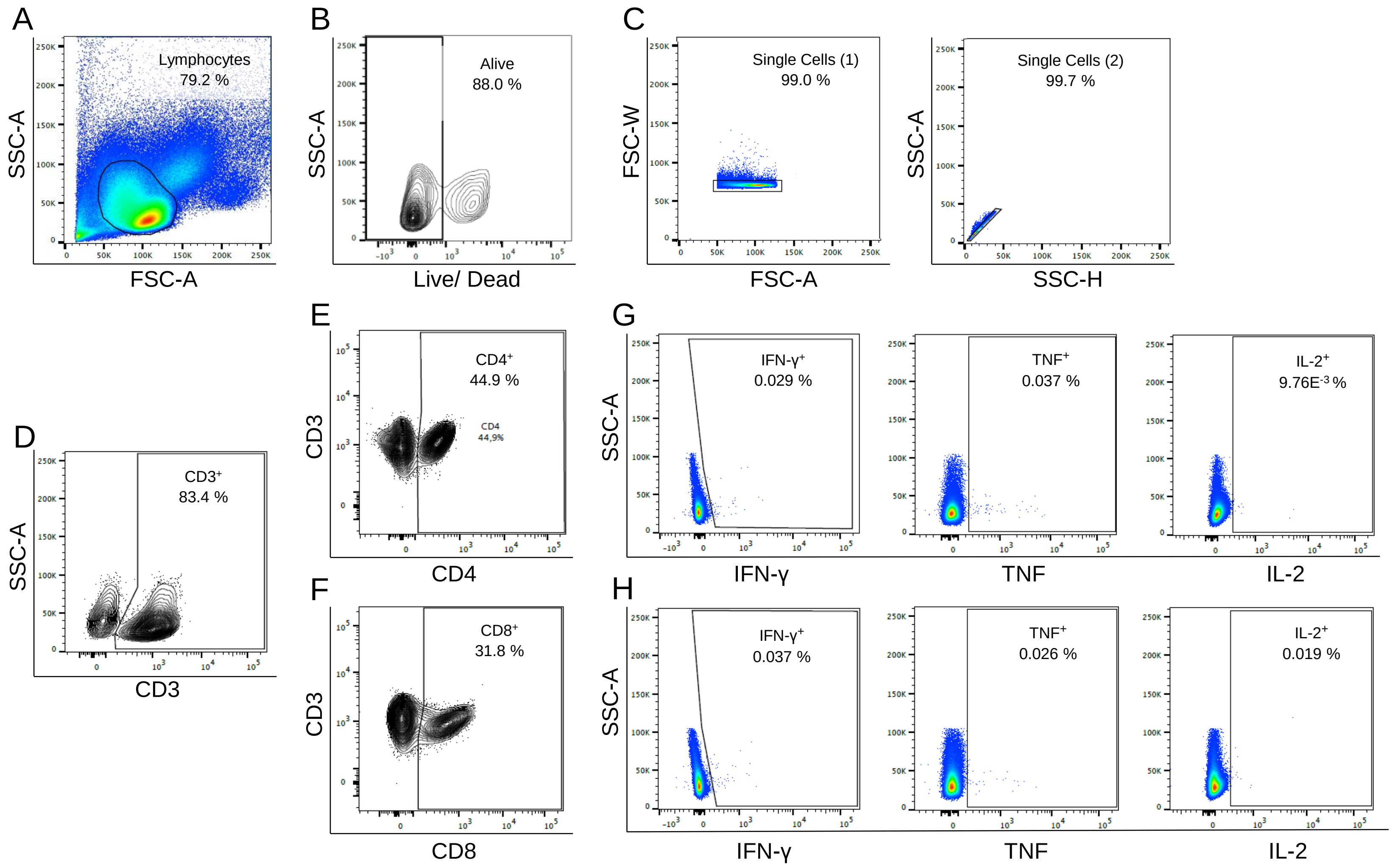


**Additional File 5 Fig. S5.** Representative Gating strategy of EBV-reactive T cells. Lymphocytes were gated based on FSC vs. SSC pseudocolor plot (A). After exclusion of dead cells (B) and doublets (C) CD3-positive lymphocytes were gated (D). Next, the CD3-positive population was gated on the expression of CD4 (E) and CD8 (F). Subsequently, CD4+ and CD8+ T cells were analysed for the expression of the intracellular cytokines IFN-γ, TNF, and IL-2 (G and H, respectively).
