# Supplementary material for: Evaluation of novel Epstein-Barr virus-derived antigen formulations for monitoring virus-specific T cells in pediatric patients with infectious mononucleosis
Source: Virol J. 2024 Jun 14;21:139. doi: 10.1186/s12985-024-02411-0 (PMC11179387; doi:10.1186/s12985-024-02411-0)
Supplement: Supplementary file 6 — Additional file 6: Table S6. Reactivity of antigen reactive CD4+ and CD8+ T cells in individual IM patients. [file 12985_2024_2411_MOESM6_ESM.pdf]

Additional file 6: Table S6: Reactivity of antigen-reactive CD4+ and CD8+ T cells in individual IM patients.

	Antigen-reactive CD4+ T cells*						Antigen-reactive CD8+ T cells*							
	BZLF1		EBNA3A		EB-VLP		BZLF1		EBNA3A		EB-VLP		PP	
**ID**	**v1**	**v2**	**v1**	**v2**	**v1**	**v2**	**v1**	**v2**	**v1**	**v2**	**v1**	**v2**	**v1**	**v2**
**1**	+	+	+	+	+	+	+	+	+	+	+	+	+	+
**2**	+	+	+	+	+	+	+	+	+	+	+	+	+	+
**3**	+	+	+	+	+	+	+	+	+	+	+	+	+	+
**4**	+	+	+	+	+	+	+	+	+	+	+	+	+	+
**5**	+	+	+	+	+	+	+	+	+	+	+	+	+	+
**6**	+	+	+	+	+	+	+	+	-	+	+	-	+	+
**7**	+	+	+	+	+	+	+	+	+	-	+	+	+	+
**8**	+	+	+	+	+	-	+	+	+	+	+	-	+	+
**9**	+	+	+	+	+	+	+	+	+	+	+	-	+	+
**10**	+	+	+	+	+	+	+	+	+	+	+	+	+	+
**11**	+	+	+	+	+	+	+	+	+	+	+	-	+	+
**12**	+	+	+	+	+	+	+	+	+	+	+	+	+	+
**13**	+	+	+	+	+	+	+	+	+	+	+	+	+	+
**14**	+	+	+	+	+	+	+	+	+	+	+	+	+	+
**15**	+	+	+	+	+	+	+	+	+	+	+	-	+	+
**16**	+	+	+	+	+	+	+	+	+	+	+	+	+	+
**17**	+	+	+	+	+	+	+	+	+	+	+	+	+	+
**18**	+	+	+	+	+	+	+	+	+	+	+	+	+	+
**19**	+	+	+	+	+	+	+	+	+	+	+	+	+	+
**20**	+	+	+	+	+	+	+	+	-	+	+	+	+	+

* antigen-specific T-cell response < LLOD highlighted in grey. v1, visit 1; v2, visit 2.
