# Supplementary material for: Evaluation of novel Epstein-Barr virus-derived antigen formulations for monitoring virus-specific T cells in pediatric patients with infectious mononucleosis
Source: Virol J. 2024 Jun 14;21:139. doi: 10.1186/s12985-024-02411-0 (PMC11179387; doi:10.1186/s12985-024-02411-0)
Supplement: Supplementary file 7 — Additional file 7. [file 12985_2024_2411_MOESM7_ESM.pdf]

Additional file 7: Table S7: Spearman correlation of frequencies of EBV-reactive CD4+ and CD8+ T cells and EBV-load in PBMC.

		v1		v2	
**Antigen reactivity**	**T-cell subpopulation**	r_s	p	r_s	p
BZLF1	CD4+	-0.095	n.s.	0.169	n.s.
	CD8+	0.108	n.s.	0.462	n.s.
EBNA3A	CD4+	0.182	n.s.	-0.250	n.s.
	CD8+	0.345	n.s.	0.060	n.s.
EB-VLP	CD4+	0.092	n.s.	0.130	n.s.
	CD8+	0.418	n.s.	0.349	n.s.
PP	CD8+	0.356	n.s.	-0.008	n.s.

v1, visit 1; v2, visit 2; r_s, Spearman correlation coefficient; n.s., not significant.
